# The readiness potential reflects intentional binding

**DOI:** 10.3389/fnhum.2014.00421

**Published:** 2014-06-10

**Authors:** Han-Gue Jo, Marc Wittmann, Thilo Hinterberger, Stefan Schmidt

**Affiliations:** ^1^Department of Psychosomatic Medicine, University Medical Center FreiburgFreiburg, Germany; ^2^Institute for Transcultural Health Studies, European University ViadrinaFrankfurt (Oder), Germany; ^3^Institute for Frontier Areas of Psychology and Mental HealthFreiburg, Germany; ^4^Department of Psychosomatic Medicine, Research Section of Applied Consciousness Sciences, University Medical Center RegensburgRegensburg, Germany

**Keywords:** sense of agency, intentional binding, readiness potential, slow cortical potential, meditation

## Abstract

When a voluntary action is causally linked with a sensory outcome, the action and its consequent effect are perceived as being closer together in time. This effect is called intentional binding. Although many experiments were conducted on this phenomenon, the underlying neural mechanisms are not well understood. While intentional binding is specific to voluntary action, we presumed that preconscious brain activity (the readiness potential, RP), which occurs before an action is made, might play an important role in this binding effect. In this study, the brain dynamics were recorded with electroencephalography (EEG) and analyzed in single-trials in order to estimate whether intentional binding is correlated with the early neural processes. Moreover, we were interested in different behavioral performance between meditators and non-meditators since meditators are expected to be able to keep attention more consistently on a task. Thus, we performed the intentional binding paradigm with 20 mindfulness meditators and compared them to matched controls. Although, we did not observe a group effect on either behavioral data or EEG recordings, we found that self-initiated movements following ongoing negative deflections of slow cortical potentials (SCPs) result in a stronger binding effect compared to positive potentials, especially regarding the perceived time of the consequent effect. Our results provide the first direct evidence that the early neural activity within the range of SCPs affects perceived time of a sensory outcome that is caused by intentional action.

## Introduction

**Figure 1:**
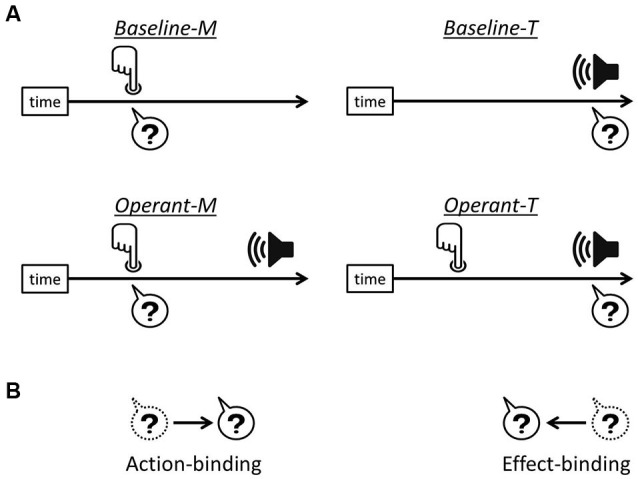
**Intentional binding paradigm. (A)** In each task, participants reported either finger movement time or the onset time of the tone, indicated by the question marks. In operant tasks, i.e., *Operant-M* and *Operant-T*, a voluntary finger movement causes the tone effect 250 ms after. **(B)** Action-binding represents the temporal attraction of finger movement time towards the tone onset in *operant-M* relative to *baseline-M*, while effect-binding represents the temporal attraction of the tone onset time towards the onset of finger movement in *operant-T* relative to *baseline-T*.

The link between a voluntary action and its consequent effect leads to the experience of controlling one’s own actions, i.e., the sense of agency. For over a decade there has been a growing interest in understanding a specific effect related to human agency, which was reported by Haggard et al. (2002) and termed “intentional binding”. They showed that when a voluntary action causes a sensory outcome, the action and the consequent effect are perceived as being closer together in time than they really are. Action-binding (the temporal attraction of action towards its consequent effect) and effect-binding (the temporal attraction of the effect towards action) were measured separately in order to investigate the intentional binding effects (see Figure 1).

The intentional binding paradigm was applied in a number of experiments to study human agency, such as self-causation (Dogge et al., 2012), action selection (Barlas and Obhi, 2013), shared actions (Strother et al., 2010), uncertainty of the effect (Wolpe et al., 2013), emotional states (Yoshie and Haggard, 2013), affective valence (Takahata et al., 2012) and beliefs in free will (Aarts and van den Bos, 2011). Although many studies have assessed how intentional binding is modulated, the underlying neural mechanisms remain relatively unexplored (Moore and Obhi, 2012). Recently, a study investigated the contribution of specific brain areas on intentional binding (Moore et al., 2010). A transient disturbance of the activity in the pre-supplementary motor area (pre-SMA) by transcranial magnetic stimulation (TMS) reduced the temporal linkage between action and the effect. This was mainly due to the fact that the sensory consequence was perceived as less shifted in time towards action. In contrast, the disruption of the contralateral sensorimotor area had no or much less influence on the temporal binding effect. These results suggested that the pre-SMA plays a crucial part in intentional binding, especially on effect-binding. Because the pre-SMA is seen as a key structure involved in conscious intention to act (Fried et al., 1991; Lau et al., 2004) and intentional binding is specifically related to intentional action (Haggard et al., 2002), this brain area is likely to be associated with the binding effects.

The intentional binding experiment starts with a self-generated action. This is similar to the Libet-type experiment which assesses preconscious brain activation (readiness potential, RP), preceding a voluntary action (Libet et al., 1983). In the literature, the RP can be divided into two components based on the scalp distribution and the slope of negative potential (Shibasaki and Hallet, 2006). The early RP starts about 2 s before a voluntary movement and consists of a prolonged and increasing negativity. This activity is localized in the bilateral pre-SMA. In contrast, the late RP has a steeper slope seen in the contralateral premotor cortex starting around −0.5 s before movement onset. Since the pre-SMA activity plays a crucial role in intentional binding (David et al., 2008; Moore et al., 2010), one can presume that the early RP might also be of importance for the temporal binding effect.

Many studies have implicated that the onset of the RP is a neural signature indicating initiation or preparation of a movement (for review, see Shibasaki and Hallet, 2006; Haggard, 2008), but recent studies suggested that the early RP is not necessarily causally related to movement preparation (Schurger et al., 2012; Jo et al., 2013, 2014). These studies rather suggest that a transient negativity of the continuously fluctuating slow cortical potentials (SCPs) facilitates the initiation of a movement in the near future. Only by averaging many single trials of this kind the early readiness potential emerges. These findings suggest that the emergence of conscious intention to act may differ in each trial as a result of differences in spontaneous brain states. Therefore, it may be more fruitful to investigate the temporal binding effect and the related brain dynamic on the level of single trials.

A number of studies have shown the positive effects of meditation on attention control and self-regulation (e.g., Jha et al., 2007; Tang et al., 2007; MacLean et al., 2010; an overview is provided in Wittmann and Schmidt, 2014). Thus, we were further interested in the effects of experience in contemplative practices on temporal attraction in an intentional binding paradigm. We hypothesized that experienced meditators would display a different temporal attraction as they are better in continuously keeping the focus of attention on the specific task conditions (Chan and Woollacott, 2007; Lutz et al., 2009; MacLean et al., 2010). Less temporal attraction in intentional binding would be indicative of less deviation from the timing of the actual event. Moreover, growing evidence of positive effects on neural systems involved in attention processes have been shown after meditation practice (Slagter et al., 2007; Lutz et al., 2009; Moore et al., 2012). Thus, different behavioral performance on intentional binding between meditators and non-meditators would be of interest regarding the question of the underlying neural mechanism of the temporal binding effect.

The aim of the present study is to investigate (i) whether the early neural activity preceding the voluntary action has an effect on intentional binding; and (ii) to explore its effect in experienced meditators by examining whether these brain correlates would be displayed differently as related to behavioral performance. In order to do so, we recorded electroencephalography (EEG) activity, while participants engaged in the intentional binding task, comparing a group of experienced meditators with matched non-meditating controls. Behavioral and electrophysiological data were analyzed on the basis of single trials.

## Methods

### Participants

Twenty experienced mindfulness meditators (seven males; mean age 40.7 years, *SD* = 7.5, range 28–50 years) volunteered for the present study. They had at least 3 years of continuous experience in regular mindfulness meditation practice and had continuous meditation practice for at least 2 h per week during the last 8 weeks. Twenty matched controls in gender, age (mean age 40.3 years, *SD* = 7.4; *p* = 0.278) and education level, were recruited. Control subjects had never attended any course of meditation practice including Yoga, Tai-Chi and similar techniques. All participants had normal or corrected-to-normal vision and had no known psychological or neurological deficits. Participants were paid 10 € per hour for taking part in the experiment. The ethics committee of the University Medical Center Freiburg approved this study and written informed consent was obtained from all participants. Participants were invited to come twice within a period of 2 weeks to two different laboratories; first for the assessment of meditation experience cognitive performance, time perception, and personality, which will be reported elsewhere, and secondly for the Libet-type tasks with EEG recording (see below the apparatus and procedure).

### Self-report measures

The Freiburg Mindfulness Inventory (FMI; Walach et al., 2006) was administered to assess the level of self-reported mindfulness. It has a two-dimensional structure with the factor “presence” referring to the ability to attend to the present moment and the factor “acceptance” referring to a non-judgmental attitude (Kohls et al., 2009). A 14-item short version has been developed which was used here.

### Apparatus and procedure

The experiment followed the procedure introduced by Haggard et al. (2002) as shown in Figure 1. Participants sat in front of a monitor and performed two baseline condition tasks (*baseline-M* and *baseline-T*) and two operant condition tasks (*operant-M* and *operant-T*) in a pseudo-random sequence. They were asked to report either the first moment of their finger movement (*m*-time) or the onset time of the tone (*t*-time). Each task contained forty trials.

In *baseline-M*, an analog clock (visual angle, 3° in diameter) was presented in the center of the screen. A clock-hand appeared after a short period (of 1–2 s delay) and started rotating clock-wise with a revolution period of 2550 ms. Participants were asked to perform a voluntary movement (pressing the left mouse button) whenever they wanted to, but not earlier than after one full rotation of the clock-hand. After the button press, the clock-hand continued rotating for a short interval (between 1–2 s) and disappeared. Participants were then asked to indicate with the mouse pointer the clock-hand position on the clock circle at the moment when they started to move their finger to press the button. The *operant-M* condition was identical to the *baseline-M* condition apart from the fact that a 500 Hz tone (presented for 100 ms) followed the button press after a delay of 250 ms. The *operant-T* condition was identical to the *operant-M* condition, but participants were asked to indicate the onset time of the tone instead of the movement onset. In the *baseline-T* condition, participants performed no voluntary button press. Instead, a tone occurred at random times between 2.6 and 7.7 s after the clock-hand started rotating. After the tone, the clock-hand continued rotating for a short interval (between 1 and 2 s) and then disappeared. Participants were then asked to indicate the clock-hand position of the tone onset.

Because of EEG recordings (see below) participants were asked to focus on the center of the clock and to refrain from eye blinking during clock-hand rotation. Presentation of the clock and collection of the response data were performed by the E-Prime 2.0 software (Psychology Software Tools, USA). Before the experiment started, participants performed two blocks of a Libet-type task, which will be reported elsewhere, and then performed a few trials of practice for each task condition.

### Electrophysiological recordings

EEG was recorded from a Quickamp amplifier using 64-channel active electrodes (Brain Products, Germany) in an acoustically and electromagnetically attenuated chamber. Ground electrode was placed on the forehead and an initial reference was placed at P9 according to the 10–20 system. Electrode impedance of all electrodes was kept under 5 kΩ. One channel electrooculography (EOG) was recorded to detect ocular artifacts. To estimate the onset of finger movement, a single axis accelerometer (1.7 g) was placed on the left mouse button to measure the exact onset time of the movement. All electrophysiological data were recorded at a sampling rate of 1000 Hz.

Pre-processing of data was performed with the help of EEGLAB version 12.02 (Delorme and Makeig, 2004). EEG records were down sampled to 250 Hz and re-referenced to linked mastoids. A band-pass filter from 0.01 to 45 Hz (zero-phase filter with −6 dB cutoff) was applied. Continuous EEG data was segmented into event-locked epochs ranging from 2.5 s before the event, either the onset of the button press or the tone, to 1 s after the event with baseline correction of the first 200 ms. Epochs affected by artifact (±100 µV) of any electrodes except ocular movement were excluded for further analysis. Remaining ocular artifacts were then corrected using independent component analysis (ICA). The trials with a button press during the first rotation of the clock-hand were also excluded. On average, 92.7% (*SD* = 8.6) epochs were analyzed.

**Figure 2:**
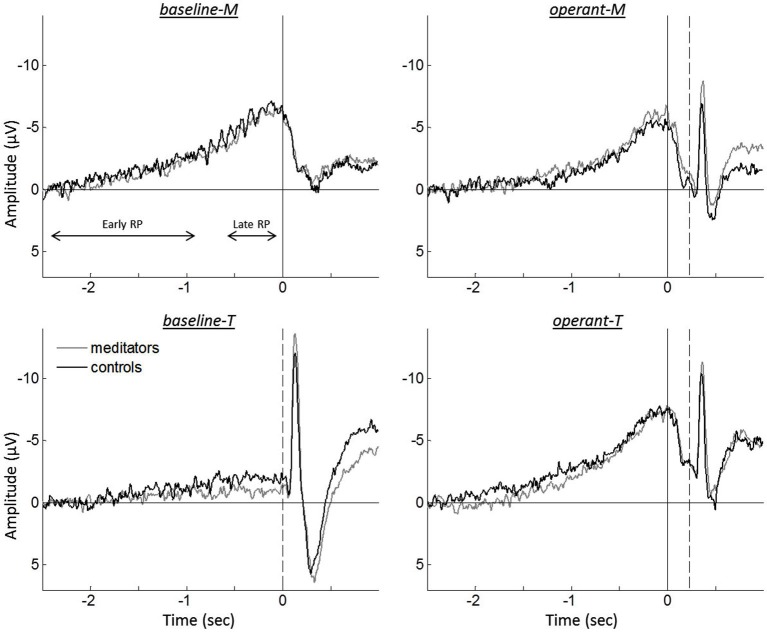
**Grand averaged event-related EEGs for meditators (gray traces) and controls (black traces), during intentional binding tasks**. Solid vertical lines represent the onset of the finger movement, while dashed vertical lines indicate the tone onset. The division for the early RP and the late RP are represented by both-sided arrows.

Event-related EEG was measured as average over the nine electrodes around Cz (FC1, FCz, FC2, C1, Cz, C2, CP1, CPz, CP2). The amplitude of the RP was then quantified calculating the mean signal during the period from −0.2 to 0 s before this button press (or before the tone onset for *baseline-T* task). Next, the RP was divided into an early and a late component (see Figure 2). We calculated separate slopes for the each part of the RP. The late RP slope was computed by dividing the amplitude difference between the mean from −0.7 to −0.5 s and the mean from −0.2 to 0 s by 0.5 s. Thereby we have divided the estimated increase of the amplitude during the last 0.5 s by its duration. For the early RP we did the analogous calculation. Since the amplitude is by definition 0 for the first 200ms due to baseline correction the overall increase was estimated by the mean amplitude from −1.0 to −0.8 s and then divided by 1.5 s, which is the duration of the early RP. In order to account for the slope of the early RP already contained in the late RP we finally subtracted the slope of the early RP from the late RP. By this procedure we can see whether there is an additional increase in the late RP compared to already ongoing trend.

To test whether ongoing potential shifts have different effects on temporal attraction, the slope of each epoch was estimated by fitting a first-order polynomial function to the average of nine electrodes before the events. According to either a negative or positive slope, each epoch was classified into either a negative or positive epoch, respectively, and then averaged for each subject.

### Data analysis

Analysis of medians rather than of simple means was applied in the present study as recommended for the Libet-type experiment (Pockett and Miller, 2007). The *m*-time and *t*-time were subtracted from the actual movement and the tone onset times, respectively. Action-binding was calculated by subtracting *m*-time during *baseline-M* from *operant-M*, and effect-binding was calculated by subtracting *t*-time during *baseline-T* from *operant-T*. Overall-binding is computed by subtracting effect-binding from action-binding. The reported times (i.e., *m*-time or *t*-time) and RP amplitudes were subject to a repeated measure ANOVA with type of reported time (*m*-time vs. *t*-time) and agency condition (baseline vs. operant) as within-subject variables, and the group (meditators vs. controls) as between-subject variables. Comparisons for matched pairs between groups were performed with paired *t*-test.

## Results

One control subject dropped out because of personal reasons. Therefore, comparison between groups was performed with 19 matched-pairs. Meditators on average had meditation experience of 10.1 years (*SD* = 6.4) and in the last 8 weeks had on average meditated for 7.6 h (*SD* = 5.2) a week.

### Self-reported data

Scores of the self-report mindfulness scale revealed significant differences between the two groups (meditators, 44.4 ± 1.1; controls, 36.5 ± 1.2; *t*_(19)_ = 4.991, *p* < 0.001), indicative of higher “acceptance” (meditators, 24.8 ± 0.8; controls, 20.1 ± 0.7; *t*_(19)_ = 4.670, *p* < 0.001) and “presence” (meditators, 19.6 ± 0.4; controls, 16.4 ± 0.6; *t*_(19)_ = 4.382, *p* < 0.001) in meditators. This result shows that meditators report themselves to be more mindful than controls.

### Behavioral data: reported times

A repeated measure ANOVA analysis revealed a significant interaction between reported time (*m*-time vs. *t*-time) and agency condition, *F*_(1,37)_ = 14.961, *p* < 0.001. To clarify this interaction, we examined the temporal binding effects for reported times, see Table 1. The reported time of the tone was shifted towards action in comparison to the baseline condition (*t*_(39)_ = −5.293, *p* < 0.001), showing effect-binding in 81.1% of the participants. In contrast, we found no significant difference in *m*-time between *baseline-M* and *operant-M* (*t*_(39)_ = 0.336, *p* = 0.739; action-binding being seen in 48.7% of the participants). That is, overall-binding was driven mainly by enhanced shift of *t*-time towards action in the *operant-T* task.

**Table 1:** **Means of reported times and temporal binding effects during intentional binding tasks**.

		Meditators	Controls	*p*-value	All participants
*m*-time	baseline	−68.35 (41.9)	−7.16 (24.0)	0.198	−38.54 (24.6)
	operant	−66.30 (25.4)	−18.34 (28.5)	0.248	−42.94 (19.2)
Action-binding		2.05 (26.8)	−11.18 (8.6)	0.639	−4.40 (14.2)
*t*-time	baseline	−32.88 (11.3)	−32.40 (18.8)	0.796	−32.64 (11.7)
	operant	−131.58 (27.6)	−122.82 (28.1)	0.856	−127.31 (19.4)
Effect-binding		−98.70 (27.4)	−90.42 (23.5)	0.774	−94.67 (17.9)
Overall-binding		100.75 (39.1)	79.24 (24.1)	0.614	90.27 (23.0)

*The m-time and t-time are obtained by subtracting the actual event time from reported time in msec (SE). Action-binding and effect-binding indicate the mean shifts in time from baseline to operant tasks for m-time and t-time, respectively. Overall-binding is the difference between effect-binding and action-binding. p-values were calculated based on nineteen matched-paired between groups*.

Notably, we found neither significant group effect nor group by task interactions (ANOVA analysis, all *p* > 0.193). Although on average we observed an earlier *m*-time in meditators than controls in both *baseline-M* and *operant-M* (see Table 1), further analysis of reported-times for all the tasks showed no difference between groups (two-tailed paired *t*-test, all *p* > 0.198).

We also conducted a one-way repeated measure ANOVA on mean waiting-time (the time from the start of a trial to the button press) with the self-generated movement tasks (*baseline-M*, *operant-M*, and *operant-T*) as a within-subject factors and group (meditators vs. controls) as between-subject variables. It revealed no task effect (*F*_(2,74)_ = 0.465, *p* = 0.630) and no group by task interaction (*F*_(2,74)_ = 2.260, *p* = 0.111). The mean waiting-times across participants were 7.21 s for *baseline-M*, 6.98 s for *operant-M*, 7.06 s for *operant-T*, and 5.06 s for *baseline-T*.

### Neurophysiologiocal data: event-related EEG

Figure 2 shows the grand averaged event-related EEG for the different tasks. A repeated measure ANOVA analysis on the RP amplitudes revealed a significant interaction between the reported time and the agency condition, *F*_(1,37)_ = 37.149, *p* < 0.001. To further test this interaction, RP amplitudes were examined for reported times (i.e., *m*-time and *t*-time). While comparison between *baseline-M* and *operant-M* revealed no differences (*baseline-M*, −6.40 µV ± 0.81; *operant-M*, −5.66 µV ± 0.73; *t*_(39)_ = −1.448, *p* = 0.156), *operant-T* showed higher amplitude as compared to *baseline-T* (*baseline-T*, −1.37 µV ± 0.49; *operant-T*, −7.19 µV ± 0.75; *t*_(39)_ = −7.330, *p* < 0.001), indicating absence of the RP in *baseline-T*. However, we found neither significant group effect nor group by task interactions (ANOVA analysis, all *p* > 0.260), displaying no difference for each task (two-tailed paired *t*-test, all *p* > 0.371). Since we found no difference between groups in both behavioral data and EEG recordings, we pooled all participants for further comparisons of the tasks.

**Figure 3:**
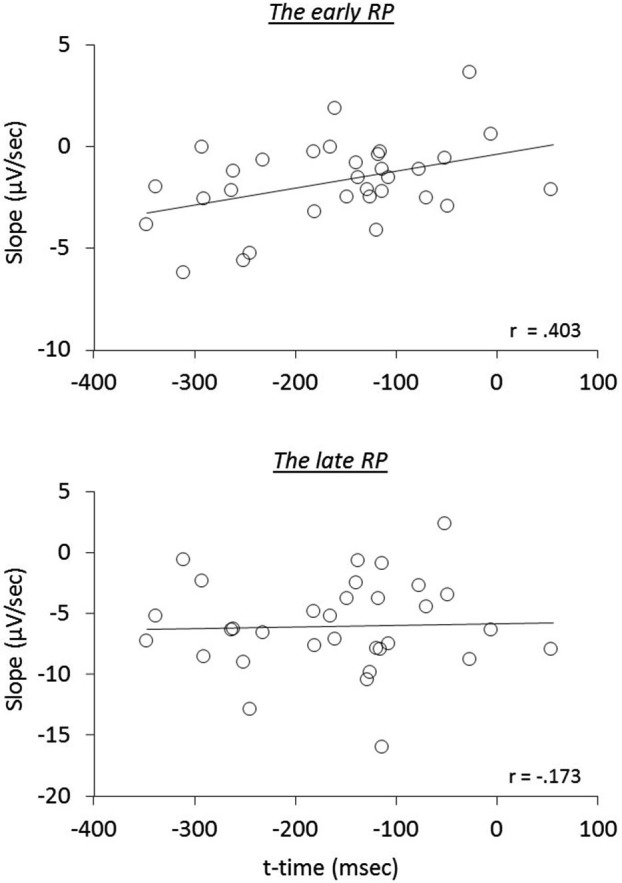
**The relation of the RP slopes to reported time of the tone during the *operant-T* task**.

We next examined the relation of reported times to RP components, i.e., whether the early neural activity before the action influences the temporal attraction. A significant correlation was found in the *operant-T* condition, namely that the more negative the early RP, the larger the shift of *t*-time towards action (*r*_(32)_ = 0.403, *p* = 0.022; seven participants, including three meditators, who showed no effect-binding were excluded), However, we did not find this correlation in the late RP (*r*_(32)_ = −0.173, *p* = 0.345; see Figure 3). Notably, no significant correlations in the other three tasks were found regarding both the early and the late RPs (all *p* > 0.215), indicating the specificity of results for the *operant-T* condition. This result suggests that the perceived time of the consequent effect is related to the neural processes of the early RP, but not with the late RP.

**Figure 4:**
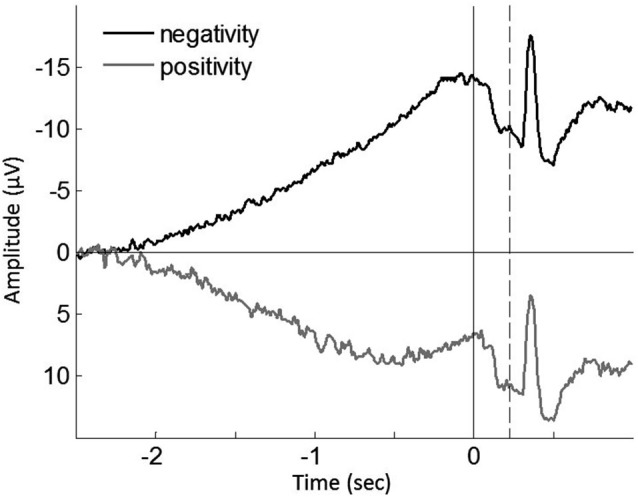
**Grand averaged ongoing negative potential (black trace) and positive potential (gray trace) during the *operant-T* task**. Solid vertical line represents the onset of the finger movement, while dashed vertical line indicates the tone onset. The shift of perceived time of the tone towards action was increased in ongoing negativity (*p* = 0.023; see the text). The grand mean of the proportion of positive epochs is 30.91% ± 2.0, which results in prolonged ongoing negativity in the early RP (see Figure 5).

**Figure 5:**
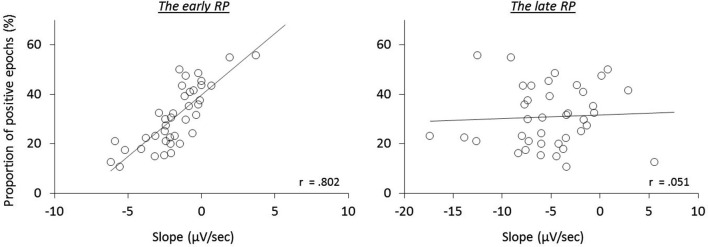
**The relation of the proportion of ongoing positive epochs to the RP slopes during the *operant-T* task**.

To further test this implication, each single trial of the individual participants was classified regarding having a negative or positive slope of the epochs, and then averaged (Figure 4). In agreement with the previous study (Jo et al., 2013), we found a significant correlation of the ratio of positive epochs with the early RP slope (*baseline-M*, *r*_(39)_ = 0.590, *p* < 0.001; *operant-M*, *r*_(39)_ = 0.644, *p* < 0.001; *operant-T*, *r*_(39)_ = 0.802, *p* < 0.001; see Figure 5), demonstrating that smaller portions of positive epochs are related to larger negative early RP. However, we observed no correlation with the late RP (*baseline-M*, *r*_(39)_ = 0.272, *p* = 0.094; *operant-M*, *r*_(39)_ = 0.224, *p* = 0.171; *operant-T*, *r*_(39)_ = 0.051, *p* = 0.758). That is, the ongoing potential shifts are specifically related to the early part of the RP. We then performed paired *t*-tests to compare reported times between ongoing negative and positive slope epochs, and found a significant difference in the *operant-T* condition (negative, −131.8 ms ± 19.2; positive, −117.8 ms ± 19.2; *t*_(39)_ = 2.370, *p* = 0.023). The shift of *t*-time towards action was larger in negative slope epochs as compared to positive ones. This supports the relation that more negative amplitudes result in stronger effect-binding. Importantly, however, we did not find the difference in the other three tasks (*baseline-M*, *t*_(39)_ = 0.079, *p* = 0.937; *operant-M*, *t*_(39)_ = −0.510, *p* = 0.613; *baseline-T*, *t*_(39)_ = −0.681, *p* = 0.500), indicating that neither the reported time of action nor the effect that is isolated with intentional action was different between negative slope epochs and positive ones. Taken together, these results provide evidence that the early neural activity affects the perceived time of a sensory outcome that is caused by intentional action.

## Discussion

In the present study, we aimed to investigate (i) the RP correlates of the intentional binding effect; and (ii) to explore these correlates in experienced meditators compared to non-meditating controls. The latter comparison did not yield any significant effect, neither in the behavioral data nor in the neurophysiological ones. On the other hand, we found that the early neural activity correlates with reported time across all participants. This finding adds to the current discussion on the underlying neural mechanisms of the sense of agency.

It is of interest that we could replicate only effect-binding but not action-binding, the latter having been shown in several other studies (Dogge et al., 2012; Barlas and Obhi, 2013; Wolpe et al., 2013). This lack of replication might be explained by the following facts: Firstly, in the present study participants were asked to report “the first moment of their finger movement” rather than the time they pressed the button. Secondly, participants were asked to gaze at the center of the clock and refrain from eye-movement, i.e., they did not trace the clock-hand movement. These two aspects have been demonstrated to significantly affect the perceived time of the events in Libet-type experiments (Pockett and Miller, 2007). Nevertheless, it is important to note that a much stronger effect-binding compared to action-binding, as found here, has been consistently shown in many other studies (e.g., Haggard et al., 2002; Moore et al., 2010, 2012; Strother et al., 2010; Aarts and van den Bos, 2011; Barlas and Obhi, 2013; Yoshie and Haggard, 2013). One explanation of this typical finding in intentional binding studies could be that participants feel a stronger sense of agency when they are asked to focus on the consequent effect rather than focusing on the action.

Regarding the RP amplitude, we found that individuals who showed a larger negative amplitude of the early RP had a higher shift of reported time towards the action (effect-binding) in the condition when participants needed to focus on the consequent effect. Consistent with this result, the ongoing shifts of the SCP within participants had a significant influence on this type of reported time, with negative slopes of the early RP being related to a larger shift towards action. Importantly, these results were only found in the *operant-T* condition, demonstrating that the early neural activity prior to movement plays a significant role in the consequent effect especially with respect to the sense of agency. Since the early RP has been related to activity in the pre-SMA (Shibasaki and Hallet, 2006), our results showing that effect-binding is specific to the early RP, but not the late RP, support the previous study by Moore et al. (2010). They reported that the transient disruption of pre-SMA using TMS showed a reduced effect-binding but not a reduced action-binding. Notably, the disruption of contralateral sensorimotor areas, which have been discussed as providing the source of the late RP, had no significant influence on temporal binding. In other words, if the pre-SMA activity had a facilitating effect, enhanced temporal attractions would be expected as a result of increased effect-binding. Overall, the present data represent the first direct evidence that the early RP plays a crucial role in the temporal attraction contributing to the effect-binding.

Notably, we found that trial-to-trial variability of the ongoing shift of SCP determined the *t*-time even when the physical condition was held constant, i.e., within the *operant-T* task. While ongoing brain fluctuation was shown to affect intrinsic motor behavior (Fox et al., 2007; Jo et al., 2014) and the early RP could reflect ongoing fluctuating SCPs (Schurger et al., 2012; Jo et al., 2013), this observation raises the possibility that temporal attraction occurs differently in dependence of the status of spontaneous brain states. Additionally, one can assume that preceding brain activity has a stronger influence on effect-binding when the action is intrinsically generated rather than triggered by external imperative stimuli. For instance, stronger effect-binding was reported in the voluntary action condition as compared to an involuntary action, though inducing the belief of self-causation could modulate the effect-binding (Dogge et al., 2012). There is strong evidence indicating that negative deflections of the spontaneous fluctuating SCPs are associated with an increasing probability of neural firing (Birbaumer et al., 1990). Therefore an action is more likely to be executed during negative shifts of the SCP. In line with this, it has repeatedly been found that a conscious intention to act could arise more likely during an ongoing negativity of the SCP, which on average results in an increased negative RP (Schurger et al., 2012; Jo et al., 2013, 2014). Within this context, the present result of the relation between the early RP and the *t*-time further suggests that if a voluntary action follows an ongoing negative potential of SCP it will more likely lead to temporal attraction of the consequent effect than with positive deflections. That is, the neural representation of conscious intention to act, ongoing negative potentials of SCP, might be associated with an enhanced sense of agency by predicting possible consequent effects of action.

There is increasing evidence that the experience of agency is generated by both predictive and postdictive processes (Synofzik et al., 2013). Regarding predictive processes, the intentional motor representation before an action is related to the experience of agency for the given action. Regarding postdictive processes, anticipation of an action’s outcome and the intention-outcome matching play the crucial role for inferring self-agency (Wegner and Wheatley, 1999). Although, many studies have repeatedly found these both effects in intentional binding (Moore and Obhi, 2012), there is still ongoing debate on whether temporal attraction is specific to intentional movement or a property of general causality perception between action and outcome (Buehner and Humphreys, 2009; Buehner, 2012). For instance, causality perception between action- and outcome-synchronized auditory signals modulated the intentional binding effect (Kawabe et al., 2013). The current finding of the relation between temporal attraction of the consequent effect and the early RP, but not with the late RP, suggests that the emergence of intention to act affects intentional binding.

In the *baseline-T* condition, we observed a slightly negative amplitude. But since there is no action preceding the tone no amplitude should be expected. A possible explanation could be that participants might have anticipated the external event. For instance, if participants learned the temporal expectancy of events, expectancy-related CNV (contingent negative variation) keeps rising until the time point of the expected event is reached even when no motor preparation is involved (Mento et al., 2013). Although the occurrence of the tones varies within an interval of 5 s, similar explanations can be applied to the result presented in the *baseline-T* condition. It should be noted, though, that the results of this task showed neither relation between event-related EEG and *t*-time nor differences in *t*-time between negative and positive epochs. Thereby one can conclude that the relation of ongoing potential shifts to *t*-time during *operant-T* are not likely due to temporal expectancy of the tone that is isolated from the sense of agency.

Although EEG recordings allow the examination of neural correlates with high temporal resolution, the temporal brain dynamics underlying human agency is not well understood (David, 2012). Several studies have observed that the brain predicts the sensory consequence of an action. The N1 amplitude was smaller in predictive sensory outcome when it was self-generated as compared to computer-generated feedback (Schafer and Marcus, 1973; Gentsch and Schütz-Bosbach, 2011; Hughes et al., 2013). Thus, N1 attenuation has been discussed as an indicator of the forward sensory model that combines self-generated motor commands and sensory information processes to predict sensory outcome. The same mechanism seems to hold in the intentional binding paradigm, as we observed sensory attenuation for the tone-evoked N1 that was self-generated (*operant-M* and *operant-T*) as compared to computer-generated (*baseline-T*; see Figure 2). However, the possibility cannot be ruled out that event-related EEG of the button press might affect the N1 amplitude.

One curious result of the present study is that the *operant-M* condition showed a lower RP amplitude as compared to the *operant-T* condition (*p* = 0.015), although both conditions contain the same action and the same consequent effect but differ in the reporting task. It can be speculated that in the different conditions participants might have changed their subjective criteria for performing a voluntary button press. Indeed, several participants reported that they tried to disregard the tone effect following their action in the *operant-M* condition. It could be that the consequent tone after the button press was seen as distractor since participants did not need to focus on it.

Regarding the group comparison, we found no differences between mindfulness meditators and controls. Meditators and controls showed the same temporal attraction in effect-binding and no action-binding. With respect to event-related EEG, no significant difference was found in the RP amplitudes for the entire tasks. We also examined whether there was any ongoing potential shift and early RP-related group differences, and found no group effect. It is possible that the selection criteria were not strong enough to recruit individuals who had sufficient experiences of mindfulness meditation. Although the FMI scores showed strong differences between groups, conceptual difficulties in the meaning of “mindfulness” and also comprehension disagreements of questionnaire items (Belzer et al., 2013) have led to doubts of whether it is possible to assess the experience of mindfulness through self-report items (Grossman, 2008). Thus, the self-report measure might not differentiate between “levels” of mindfulness but differences found here might describe different levels of conceptual knowledge. Another possible explanation is that meditators may have performed the task by focusing on their perceived time rather than the actual event time. For instance, we observed earlier *m*-time in meditators than controls in both *baseline-M* and *operant-M* conditions (see Table 1), though it revealed no significant difference. It might be that meditators reported the moment of “intention” to act, which is shortly before the actual movement onset. Therefore, further work may concern the possible divergences of subjective criteria, whether focusing on perceived-events or actual events.

In conclusion, our results do not support the hypothesis that mindfulness meditators would display different performance on the intentional binding paradigm as compared to controls. However, the present findings of the early RP correlates with the temporal attraction shed light on the underlying neural mechanism of human agency. Our results suggest that the early neural activity within the range of ongoing potential shifts affects the perceived time of the sensory outcome that is caused by intentional action.

## Conflict of interest statement

The authors declare that the research was conducted in the absence of any commercial or financial relationships that could be construed as a potential conflict of interest.
